# M3R拮抗剂对人小细胞肺癌增殖、凋亡及粘附的影响

**DOI:** 10.3779/j.issn.1009-3419.2016.03.01

**Published:** 2016-03-20

**Authors:** 文婷 吴, 淑香 张, 彩红 胡

**Affiliations:** 1 750004 银川，宁夏医科大学 Ningxia Medical University Yinchuan 750004, China; 2 750004 银川，宁夏医科大学总医院呼吸与危重症学科 Department of Respiratory and Critical Care Medicine, Affiliated Hospital of Ningxia Medical University, Yinchuan 750004, China

**Keywords:** 毒蕈碱胆碱受体3, 肺肿瘤, 细胞增殖, 细胞凋亡, 细胞粘附, Muscarinic receptor 3, Lung neoplasms, Cell proliferation, Cell apoptosis, Cell adhesion

## Abstract

**背景与目的:**

研究表明毒蕈碱胆碱受体3（muscarinic receptor 3, M3R）在多种肿瘤的发生、发展中发挥重要的作用。本研究旨在探讨M3R在人小细胞肺癌（small cell lung cancer, SCLC）细胞株SBC3的表达，M3R拮抗剂对细胞增殖、凋亡及粘附的影响。

**方法:**

体外培养SBC3细胞，RT-PCR和Western blot检测M3R的表达。MTT法及流式细胞法检测M3R拮抗剂（4-diphenylacetoxy-N-methylpiperidine methiodide, 4-DAMP）对细胞增殖和凋亡的影响。流式细胞法检测细胞整合素的表达及碘化乙酰胆碱（acetylcholine iodide, Ach）和4-DAMP对整合素表达的影响。纤维结合蛋白（Fn）包被的96孔板用以研究Ach、4-DAMP及整合素抗体对细胞粘附的作用。

**结果:**

SBC3细胞表达M3R，4-DAMP浓度依懒性抑制细胞增殖。与对照组比较，10^-4^ M 4-DAMP能够明显地增加SBC3细胞凋亡。SBC3细胞表达αvβ1和α5β1整合素，10^-4^ M Ach刺激细胞粘附（*P*＜0.01）的作用几乎被10^-5^ M 4-DAMP、5 μg/mL抗-β1抗体或抗-αv和α5抗体完全阻断（*P*＜0.01），但Ach及4-DAMP不影响αv、α5和β1的表达水平。

**结论:**

SBC3细胞表达M3R，M3R拮抗剂能抑制细胞的增殖并促进凋亡。其抑制粘附的作用是通过抑制细胞含β1的整合素（αvβ1和α5β1）的功能实现的。

## Abstract (en)

**Background and objective:**

Studies have shown that muscarinic receptors 3 (M3R) plays key roles in regulating tumorigenesis and tumor growth. The aim of this study is to investigate the expression of M3R in human small cell lung cancer (SCLC) cell line SBC3 and to explore the effect of M3R antagonist on cell proliferation, apoptosis and adhesion.

**Methods:**

SBC3 cells were cultured *in vitro*. RT-PCR and Western blot were used to detect the expression of M3R in SBC-3. MTT assay and flow cytometry were used to determine the effects of M3R antagonist 4-diphenylacetoxy-N-methylpiperidine methiodide (4-DAMP) on the proliferation and apoptosis of SBC-3 cells. Flow cytometry was used to detect the integrin expression in SBC3 and alteration of integrin expression after treating the cells with cholinergic receptor agonist acetylcholine iodide (acetylcholine iodide, Ach) and 4-DAMP. Fibronectin (Fn) coated 96-well plates were used to detect the effect of Ach, 4-DAMP and anti-integrin antibody on cell adhesion.

**Results:**

M3R was expressed in SBC3 cells. 4-DAMP significantly inhibited SBC3 cells proliferation in a dose-dependent manner. 4-DAMP promoted cell apoptosis at a concentration of 10^-4^ M of 4-DAMP compared with control. αvβ1 and α5β1 integrin were expressed in SBC3 cells. 10^-4^ M Ach stimulated cell adhesion toward Fn significantly (*P* < 0.01). This effect was completely abrogated by 10^-5^ M 4-DAMP, 5 μg/mL anti-β1 integrin antibody or anti-αv and anti-α5 integrin antibodies (*P* < 0.01) and partially abrogated by 5 μg/mL anti-αv or anti-α5 integrin antibody (*P* < 0.05). But Ach and 4-DAMP did not alter Fn receptor (αvβ1 or α5β1 integrin) expression.

**Conclusion:**

M3R is expressed in SBC3 cell. The M3R antagonist inhibits SBC3 cell proliferation, adhesion and enhances cell apoptosis. M3R antagonist inhibiting SBC3 cell adhesion is presumably modulated by functional alteration of β1 containing integrins (αvβ1 and α5β1), but not by any variation in their expression.

毒蕈碱胆碱受体（muscarinic receptor, MR）是G蛋白偶联受体家族成员之一，共5个亚型M1R-M5R。近年来发现：M3R激动剂能够刺激黑色素瘤、胰腺癌、乳腺癌、卵巢癌、前列腺癌、脑癌的细胞增殖；同时其拮抗剂很可能抑制肿瘤的生长^[1]^。肿瘤的发生、发展与细胞增殖过度、凋亡受抑以及在其他脏器的粘附、转移有关。本实验的目的是首先观察体外培养小细胞肺癌（small cell lung cancer, SCLC）细胞株表达M3R，进而研究M3R拮抗剂对SCLC细胞株增殖、凋亡及粘附的影响，可为M3R拮抗剂在SCLC辅助治疗方面提供实验依据。

## 1 材料和方法

### 1.1 材料

细胞株：人SCLC细胞株SBC3购自日本JCRB细胞库。试剂：RPMI-1640培养基（Hyclone）；胎牛血清（Gibco）；Trizol（Invitrogen）；First Strand cDNA Synthesis kit（Thermo）；全蛋白提取试剂盒（凯基）；Bradford蛋白浓度测定试剂盒（碧云天）；兔抗人M3R多克隆Ⅰ抗（Abcam）；β-actin单克隆Ⅰ抗（CST）；抗兔辣根过氧化物酶标记Ⅱ抗（CST）；增强化学发光（ECL）试剂（Thermo）；M3R拮抗剂4-DAMP（Sigma）；胰岛素-转铁蛋白-锌（ITS）（Gibco）；四甲基偶氮唑（MTT）（Sigma）；Annexin-FITC/PI凋亡试剂盒（贝博）；鼠抗人αv、α2、α5、α6、β1和β3整合素单克隆Ⅰ抗（Chemicon公司）；抗鼠IgG异硫氰酸荧光素（FITC）标记Ⅱ抗（Chemicon公司）；Propidium iodide（PI）（Sigma公司）；人纤维结合蛋白（Fn）（Roche公司）；牛血清白蛋白（BSA）（Sigma公司）；胆碱受体激动剂碘化乙酰胆碱（acetylcholine iodide, Ach）（Wako公司）。

### 1.2 细胞培养

SBC3细胞培养于含10%FBS，1%青链霉素混合液的RPMI-1640培养液中，置于37 ℃、5%CO_2_培养箱内常规培养。

### 1.3 RT-PCR检测M3R mRNA表达

按Tirzol试剂盒说明书提取细胞总RNA，用Dnase Ⅰ去除总RNA中残留的基因组DNA，参照逆转录反应试剂说明完成cDNA的合成，所得产物进行PCR反应。引物（上海生工）如下：β-actin引物上游5’-AGAAAATCTGGCACCACACC-3’，β-actin引物下游5’-AGGAAGGAA GGCTGGAAGAG-3；M3R引物上游5’-TGGAACAACAATGATGCT GC-3’，M3R引物下游5’-CCTTTTCCGCTTAGTGATCTG-3’。扩增条件：预变性94 ℃ 3 min，94 ℃ 30 s，60 ℃ 30 s，72 ℃ 45 s共30个循环，最后72 ℃ 7 min。PCR产物行1%琼脂糖凝胶电泳，紫外线照相。

### 1.4 Western blot检测M3R蛋白表达

按全蛋白提取试剂盒说明书提取蛋白。用酶标仪测蛋白浓度后定量。加热变性后，每样本100 μg行SDS-PAGE电泳，后电转移至NC膜上。5%脱脂奶粉TBST封闭1 h，洗膜，后加兔抗人M3R多克隆Ⅰ抗（1:500）兔抗人β-actin Ⅰ抗（1:1, 000）室温孵育1 h，后4 ℃过夜，复温1 h。洗膜后加入HRP标记的抗兔Ⅱ抗（1:1, 500）室温孵育1 h，加ECL显影剂，暗室曝光显影^[2]^。

### 1.5 MTT法检测M3R拮抗剂对细胞增殖的影响

取对数生长期的细胞，每孔2, 000个细胞种植于96孔板中。细胞经24 h贴壁后弃去上清，换1%ITS无血清培养基同步化一天后加药，共分为2组：①正常对照组：每孔加入100 μL 1%ITS+1%BSA的培养基；②4-DAMP组；每孔加入100 μL含有不同浓度的4-DAMP的1%ITS+1%BSA培养基，使4-DAMP终浓度分别为10^-4^ M、10^-5^ M、10^-6^ M、10^-7^ M。培养72 h后向每孔加10 μL MTT。在培养箱孵育4 h后，弃掉上清，加入200 μL DMSO，置摇床震荡10 min，使结晶物充分溶解。后在570 nm波长的酶标仪下读出OD值。计算药物对细胞的抑制率。抑制率=（1-实验组OD值/对照组OD值）×100%。以4-DAMP浓度的对数为横坐标，抑制率为纵坐标作图并拟合抑制曲线，50%抑制率所对应的化合物浓度即为IC_50_值。每组不同浓度药物处理的细胞样本加5个复孔，所有实验至少重复3次^[3]^。

### 1.6 Annexin V-FITC/PI流式凋亡试剂盒检测细胞凋亡

取对数生长期的细胞，2.0×10^5^个细胞种植于60 mm培养皿中。细胞经24 h贴壁后弃去上清，加1%ITS无血清培养基同步化一天后加药。分正常对照组和10^-4^ M 4-DAMP组。细胞培养72 h后收集上清。用不含EDTA胰蛋白酶消化细胞，离心，用PBS制备单细胞悬液洗涤两次并计数。使各样本细胞浓度大约为1×10^6^/mL细胞。加入400 μL的1×Annexin V结合液重悬细胞；避光条件下加入5 μL Annexin V-FITC染色液和10 μL PI染色液后立即用流式细胞仪（BD公司Accuri C6）检测，用BD Accuri C6软件进行分析。

### 1.7 M3R对细胞粘附的影响

流式细胞法检测SBC3细胞整合素的表达：观察细胞生长状态良好，0.05%EDTA收集细胞，PBS液洗涤2遍，调整细胞浓度为5×10^6^/mL。分别取100 μL细胞液，加入抗人αv、α2、α5、α6、β1和β3整合素抗体，浓度为10 μg/mL，4 ℃孵育1 h。PBS液洗2遍，分别加入抗鼠IgG FITC抗体，浓度为10 μg/mL，4 ℃孵育30 min。细胞再次用PBS液洗涤2遍后，200 μL PBS液重悬，充分混匀后加入PI，浓度为10 μg/mL。FACScan^TM^（Becton-Dickinson，美国）检测分析整合素的表达。其次，按上述方法收集细胞、调整细胞浓度后，加入10^-5^ M 4-DAMP，作用30 min后加入10^-4^ M Ach，37 ℃、5%CO_2_的培养箱中培养6 h，流式细胞法观察整合素αv、β1和α5的表达在上述药物作用后有无改变。

体外细胞黏附实验：PBS液配制的20 μg/mL Fn和10 mg/mL BSA分别包被96孔板（Corning Incorporated，美国），4 ℃放置过夜。第2日，PBS液洗3遍，各孔加入10 mg/mL BSA 100 μL，37 ℃培养箱放置1 h，再次用PBS液洗2遍。0.05%EDTA收集生长状态良好的细胞，1%ITS RPMI-1640培养液洗涤细胞1遍并重悬细胞。试验组分为6组，10^-5^ M 4-DAMP、5 μg/mL抗人αv、β1、α5或αv和α5整合素抗体分别加入其中5组细胞，30 min后加入10^-4^ M Ach，第6组只加入10^-4^ M Ach。各组细胞以5×10^5^个/孔分别种植于BSA和Fn包被好的96孔板，BSA和Fn包被的孔中加入未经任何药物处理的细胞分别作为阴性和阳性对照。37 ℃、5%CO_2_培养箱中培养。2 h后，离心并PBS液清洗各孔以去除没有粘附的细胞。显微镜下计数5个高倍视野（400倍）的细胞数，求平均值计作粘附细胞数。每组细胞每板加5个复孔，所有试验至少重复3次。

### 1.8 统计学方法

应用SPSS 11.5软件统计分析。实验数据用均数±标准差（Mean±SD）表示，组间比较采用两样本*t*检验，以*P*＜0.05为差异有统计学意义。

## 2 结果

### 2.1 M3R在SBC3的表达

RT-PCR电泳结果所示：以不加模板作为阴性对照，未见条带，说明PCR体系无污染。加入模板的可见特异性M3R mRNA（432 bp）目的条带的表达。Western blot结果如图 1B所示：SBC3细胞可检测出分子量约为66 kDa的M3R蛋白的表达（图 1）。

**1:**
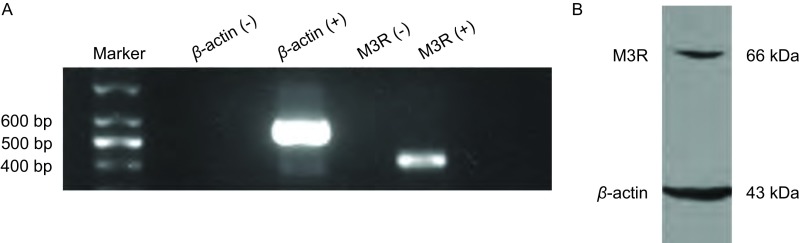
SBC3细胞株表达M3R。A：PT-PCR检测M3R mRNA在SBC3中的表达。B：Western blot检测M3R蛋白在SBC3中的表达。 SBC3 cells expressed M3R. A: Expression of M3R mRNA in SCLC cell line SBC3. B: Western blot detected the protein of M3R in SBC3 cells.

### 2.2 M3R对SBC3增殖的影响

4-DAMP浓度依赖性地抑制SBC3细胞的增殖。实验结果显示，药物对细胞的IC_50_为22.8 μM（图 2）。

**2:**
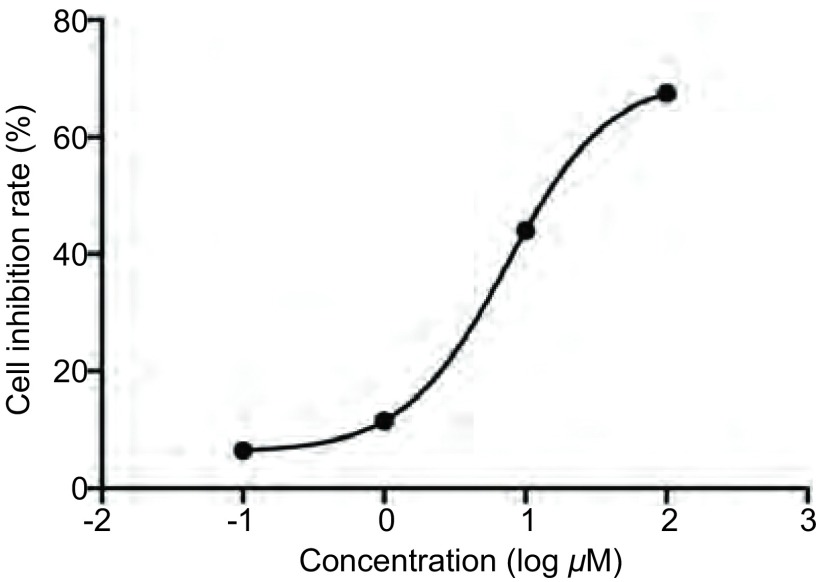
M3R受体拮抗剂4-DAMP浓度依懒性抑制细胞增殖。 Muscarinic receptor antagonist 4-DAMP significantly inhibited SBC3 cells proliferation

### 2.3 M3R拮抗剂对SBC3凋亡的影响

为检测4-DAMP对SBC3细胞的凋亡影响。我们采用了Annexin-V-FITC/PI双染法进行检测。与对照组相比，10^-4^ M 4-DAMP能够明显地增加SBC3细胞凋亡。正常对照组早期凋亡率为（1.56±0.11）%，药物组早期凋亡率为（6.46±1.50）%，差异有统计学意义（*P*=0.005），对照组总凋亡率为（4.43±0.15）%，药物组总凋亡率为（16.03±1.27）%，差异有统计学意义（*P*＜0.001）（图 3，图 4）。

**3:**
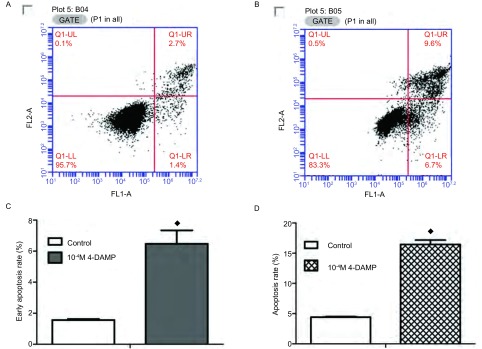
采用Annexin V-FITC/PI流式凋亡试剂盒检测4-DAMP对SBC3细胞凋亡的影响。A：阴性对照组，未加任何干扰因素。B：终浓度为10^-4^ M 4-DAMP处理组。C：与正常组相比，4-DAMP处理组的早期凋亡率高于正常组。D：与正常组相比，4-DAMP组总凋亡率明显高于正常组（◆*P*＜0.01）。 4-DAMP promoted SBC3 cell apoptosis. A: The cells treated without drug was negative control. B: The cells treated with 4-DAMP (10^-4^ M). After harvested, cells were stained with Annexin-v-FITC and PI. C: Representative analysis of flow cytometry for the detection of early apoptosis in SBC3 cells stimulated with and without 4-DAMP. D: Compared with control. The ratio of apoptosis was higher after exposure to 4-DAMP for 72 h (◆*P* < 0.01 *vs* control)

**4:**
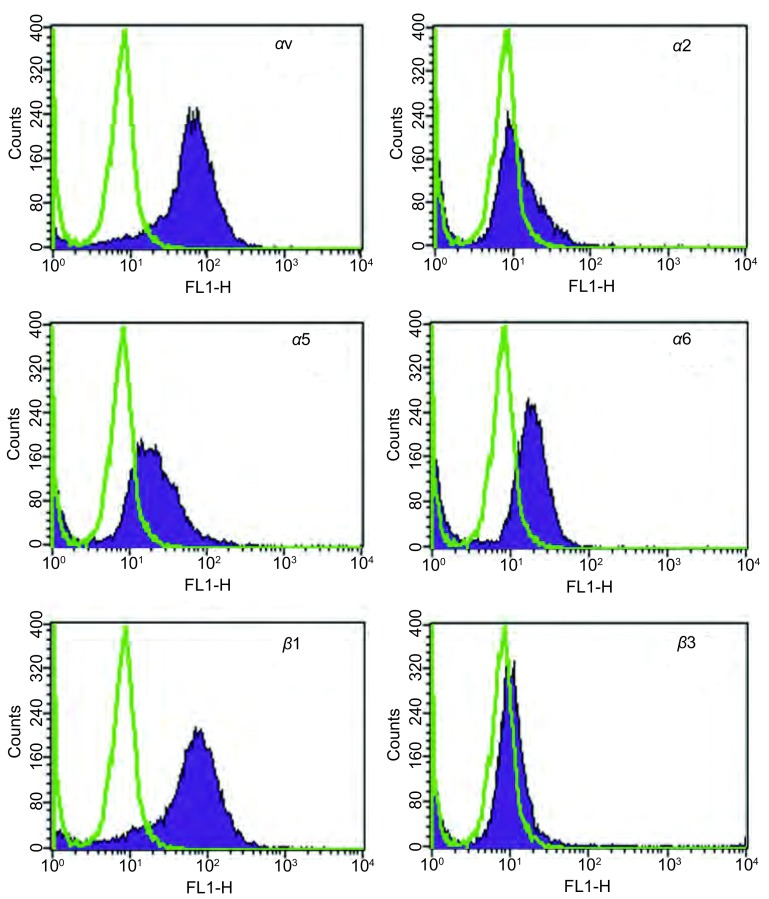
流式细胞法检测SBC3整合素表达SBC3主要表达*α*v和*β*1整合素，少量表达*α*5整合素。 Flow cytometry detected the expression of integrin. The *α*v and *β*1 integrin were predominantly expressed in the SBC cells and *α*5 was minor.

### 2.4 M3R对SBC3粘附的影响

整合素在SBC3的表达：本研究检测了SBC3细胞上αv、α2、α5、α6、β1和β3整合素的表达。SBC3细胞主要表达αv和β1整合素，同时也少量表达α5整合素。这个结果提示，SBC3细胞主要表达αvβ1，其次是α5β1整合素（图 4）。

M3R对SBC3细胞上整合素αv、β1和α5表达的影响：流式法进一步检测经4-DAMP和Ach处理过的SBC3细胞上αv、β1和α5整合素表达情况。SBC3细胞上αv、β1或α5整合素的表达水平较药物处理前没有发生变化（图 5）。

**5:**
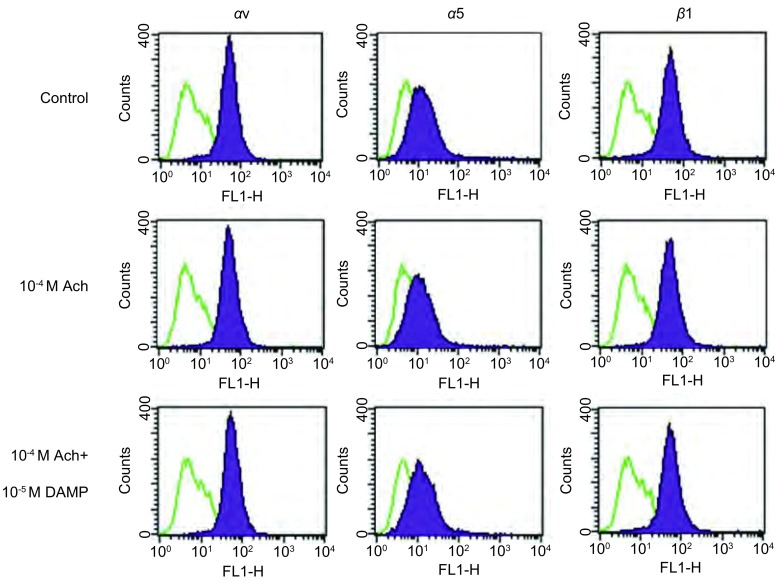
Ach和4-DAMP对SBC3细胞表面整合素表达的影响。10^-4^ M Ach、10^-4^ M Ach+10^-5^ M 4-DAMP不改变SBC3细胞表面*α*v、*β*1及*α*5整合素的表达水平。 Ach and 4-DAMP did not have an effect on cells integrin expression. *α*v, *α*5 and *β*1 expression was not altered after the cells were treated with 10^-4^ M Ach or 10^-4^ M Ach+10^-5^ M 4-DAMP.

M3R对SBC3细胞黏附的调节作用：SBC3细胞几乎不能粘附于BSA包被的96孔板，但可以粘附于Fn包被的96孔板。同时，10^-4^ M Ach明显地刺激SBC3细胞对Fn的粘附（*P*＜0.01），Ach的刺激作用几乎完全被10^-5^M的M3R拮抗剂4-DAMP所阻断（*P*＜0.01），部分被5 μg/mL抗-αv或抗-α5整合素抗体所阻断（*P*＜0.05），几乎完全被5 μg/mL抗-β1整合素抗体或5 μg/mL抗-αv和抗-α5整合素抗体所阻断（*P*＜0.01）。图 6B所示为10^-5^M 4-DAMP对Ach刺激的SBC3细胞粘附抑制作用的显微镜下所见。图 7B所示为整合素抗体对Ach刺激的SBC3细胞粘附抑制作用的显微镜下所见（图 6，图 7）。

**6:**
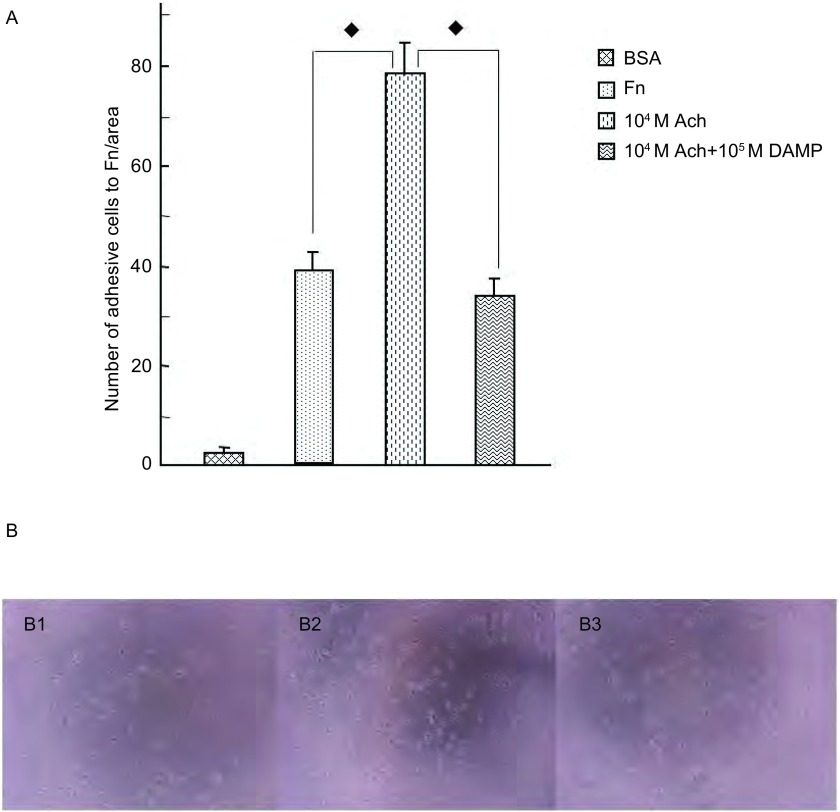
M3R对SBC3细胞粘附的调节作用。A：10^-4^ M Ach明显地增强SBC3细胞对Fn的粘附，10^-5^ M 4-DAMP完全阻断Ach对细胞粘附的刺激作用（◆*P*＜0.01）。B：4-DAMP对Ach刺激的SBC3细胞粘附作用显微镜下表现（400×）。B1：Fn；B2：10^-4^ M Ach；B3：10^-5^ M 4-DAMP+10^-4^ M Ach。 Role of M3R on cells adhesion. A: 10^-4^ M Ach promoted cells adhesion towards Fn, but fully blocked by 10^-5^ M 4-DAMP. B: The influence of M3R on cells adhesion were shown under microscope (400×). B1: Fn; B2: 10^-4^ M Ach; B3: 10^-5^ M 4-DAMP+10^-4^ M Ach.

**7:**
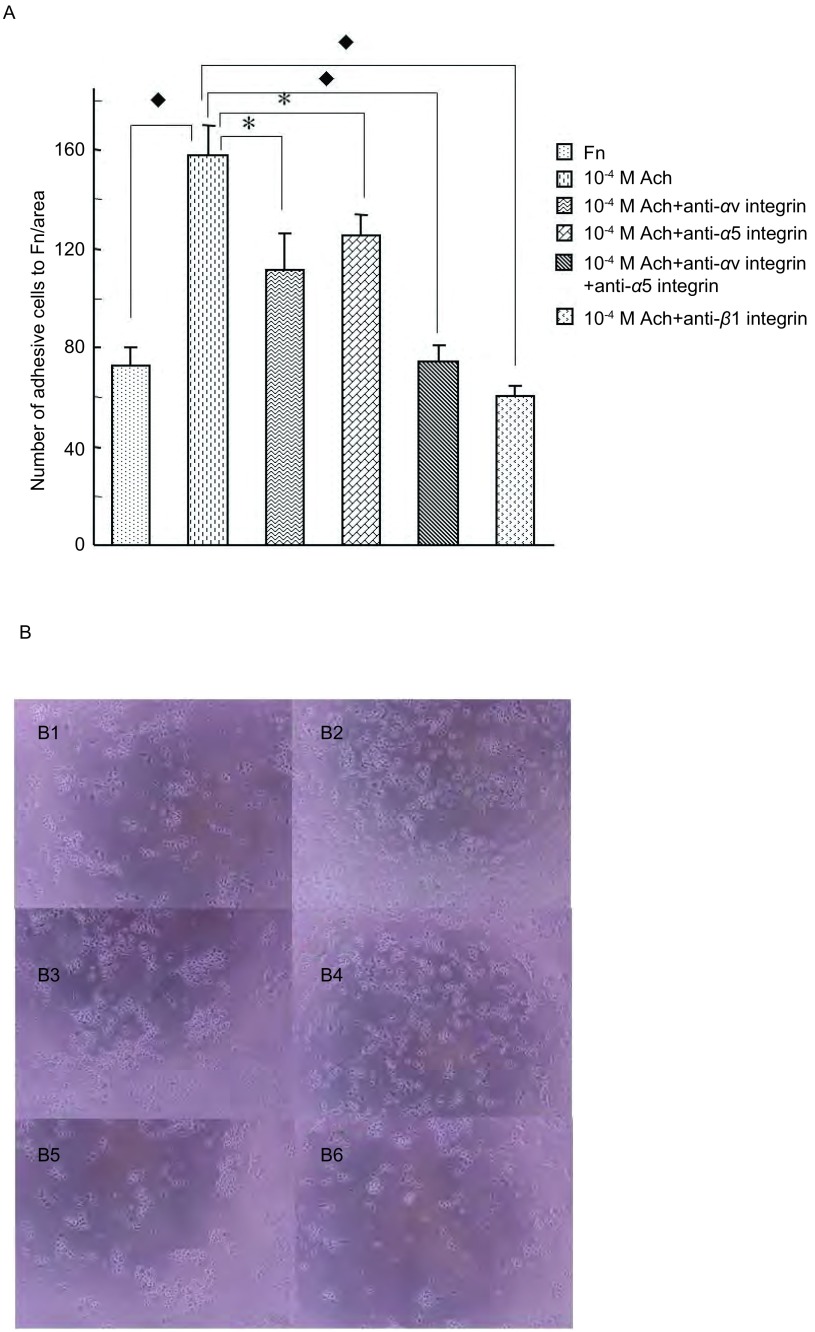
整合素对SBC3细胞粘附的调节作用。A：*α*v或*α*5整合素抗体部分阻断Ach对细胞粘附的刺激作用（^*^P＜0.05），*α*v和*α*5或*β*1整合素抗体完全阻断Ach对细胞粘附的刺激作用（◆*P*＜0.01）。B：整合素抗体对Ach刺激的SBC3细胞粘附作用显微镜下表现（400×）。B1：*Fn*；*B*2：10^-4^ M Ach；B3：10^-4^ M Ach+5 *μ*g/mL anti-*α*v整合素；B4：10^-4^ M Ach+5 *μ*g/mL anti-*α*5整合素；B5：10^-4^ M Ach+5 *μ*g/mL anti-*α*v+5 *μ*g/mL anti-*α*5整合素；B6：10^-4^ M Ach+5 *μ*g/mL anti-*β*1整合素。 Integrin regulated cell adhesion. A: anti-*α*v integrin antibody or anti-*α*5 integrin antibody could partially block the effect of cell adhesion stimulated by Ach, anti-*α*v integrin combine with anti-*α*5 integrin or anti-*β*1 integrin fully blocked the influence of Ach on cell adhesion. B: The photos under microscope show the role of integrin antibody have an inhibitory effect of cell adhesion stimulated by Ach (400×). B1: Fn; B2: 10^-4^ M Ach; B3: 10^-4^ M Ach+5 *μ*g/mL anti-*α*v integrin; B4: 10^-4^ M Ach+5 *μ*g/mL anti-*α*5 integrin; B5: 10^-4^ M Ach+5 *μ*g/mL anti-*α*v integrin+5 *μ*g/mL anti-*α*5 integrin; B6: 10^-4^ M Ach+5 *μ*g/mL anti-*β*1 integrin.

## 3 讨论

Wessler等^[4]^提出非神经元性胆碱能系统的概念，非神经元性Ach从细胞中分泌出来，通过自分泌或旁分泌机制，作用于自身或邻近细胞上的尼古丁胆碱受体（nicotinic receptor, NR）和MR，调节细胞的基本功能，如基因表达、扩增、分化、细胞骨架的形成、细胞间接触等，越来越多的研究发现M3R和肿瘤的发生、发展关系密切。我们研究发现SCLC细胞株SBC3表达M3R，M3R拮抗剂4-DAMP抑制SBC3的增殖、粘附并促进细胞的凋亡。

Williams^[5]^发现，胆碱受体激动剂卡巴胆碱减少SCLC细胞DNA的合成继而抑制细胞增殖；而Song^[6]^的研究发现，胆碱受体激动剂Ach刺激SCLC的增殖，选择性的M3R拮抗剂能够抑制SCLC的增殖。我们的结果与Song的结果一致。这种不同的结果在其他文献中也有报道。乳腺癌细胞LM2.LM3表达MRs，卡巴胆碱在短时间内促进细胞增殖，但在长时间内却能抑制细胞增殖^[7]^。小鼠成纤维细胞NIH3TS处于不同生长环境中，卡巴胆碱既有促进细胞增殖的作用，也有抑制细胞增殖的作用，作者认为这种矛盾的结果与细胞类型、受体水平、实验条件、观察时间等因素有关^[8]^。我们认为细胞是向恶性转化还是向良性转化是由多个因素决定的过程，需要更多的细胞株通过不同的实验方法去验证。

MRs和细胞凋亡的关系的研究主要集中在神经元细胞，有研究^[9]^发现与Gq亚型单位偶联的M1R、M3R、M5R活化后能够保护细胞发生DNA损伤从而抑制凋亡的发生，而与Gi亚单位偶联的M2R、M4R没有这样的特性。Budd等^[10]^报道转染MRS的中国仓鼠卵巢细胞，MR激动剂卡巴胆碱抑制依托泊苷诱导的细胞凋亡，这种作用被阿托品所阻断。有研究^[11]^发现，慢性阻塞性肺疾病患者外周血T淋巴细胞表面的M3R水平明显升高，M3R拮抗剂噻托溴铵能够促进T淋巴细胞的凋亡。目前，尚没有MRS与SCLC凋亡的相关研究，我们在倒置显微镜下观察到4-DAMP对SBC3细胞凋亡有明显影响，同时通过流式细胞仪检测了4-DAMP对SBC3细胞凋亡的影响。4-DAMP能够明显促进SBC3细胞的凋亡。4-DAMP浓度为10^-4^ M时，正常活细胞比例降低；早期凋亡、晚期凋亡比例明显升高；并且晚期凋亡比例高于早期凋亡比例，与对照组相比，有统计学意义。肿瘤的发生，发展与细胞增殖过度，凋亡抑制有关，细胞凋亡对肿瘤起负调控作用。目前细胞凋亡途径有两条：死亡受体途径和线粒体途径，关于M3R对SCLC凋亡的具体机制，需要我们进一步深入研究。

细胞粘附于细胞外基质（extracellular matrixc, ECM）的能力和肿瘤的转移和扩散密切相关。在Quigley^[12]^的研究中发现，卡巴胆碱诱导SCLC细胞株SCC-9粘附于Ⅳ型胶原，非选择性MR拮抗剂阿托品R和抗-β1整合素抗体都能够阻断卡巴胆碱的这种作用。目前尚没有M3R对SCLC粘附作用的研究。整合素是调节细胞与细胞之间、细胞与细胞外基质间连接的一种主要的跨膜受体，是由α和β亚单位组成的异源二聚体。在哺乳动物，目前发现共有19个α和8个β亚单位^[13]^。Fn是ECM中一种糖蛋白，细胞表面的整合素和Fn结合，调节细胞的粘附、迁移和侵袭^[14]^。本研究检测了整合素αv、α2、α5、α6、β1和β3在SBC3细胞的表达，发现SBC3细胞主要表达αv和β1，也有少量α5的表达。说明SBC3细胞主要表达αvβ1，其次是α5β1整合素。α5β1整合素是经典的Fn受体。αvβ1最早在神经肿瘤细胞株IMR32中发现^[15]^，之后在SCLC和鳞癌中都发现了αvβ1的表达^[16, 17]^。αvβ1的一个主要功能也是调节细胞向Fn的粘附和迁移。本研究发现，SBC3细胞几乎不能粘附在10 mg/mL BSA包被的96孔板中，20 μg/mL Fn包被的96孔板中粘附的细胞明显增多，这间接说明在SBC3细胞α5β1和αvβ1是有功能的整合素。10^-4^ M外源性Ach刺激细胞对Fn的黏附，10^-5^ M 4-DAMP几乎完全阻断了外源性Ach的这种作用，说明Ach是通过作用于M3R刺激细胞粘附的。另外，抗-β1整合素抗体或抗-αv和抗-α5整合素抗体也能够完全阻断外源性Ach刺激的SBC3细胞对Fn的粘附，抗-αv或抗-α5整合素抗体部分地阻断Ach刺激的SBC3细胞对Fn的粘附，这表明，M3R调节SBC3细胞粘附于Fn的作用是通过含有β1亚单位的整合素受体（αvβ1和α5β1）实现的。我们推测，Ach或许能够增强细胞表面整合素受体的表达从而刺激细胞粘附于Fn，而且这种作用会被4-DAMP所阻断。然而，和我们的推测相反，外源性的Ach、4-DAMP都不能改变SBC3细胞表面αv、α5或β1整合素的表达水平。这些结果表明M3R通过含有β1亚单位（αvβ1和α5β1）的整合素调节细胞的粘附是通过改变整合素的活性和功能实现的，而不能改变细胞表面整合素的表达水平。我们认为Ach作用于M3R，通过增加含有β1亚单位整合素的活性，刺激细胞在ECM中的粘附和运动，能够加速SCLC的转移，M3R拮抗剂阻断Ach和受体的结合，从而抑制SCLC的转移。当然，在体内，会不会得到同样的结果，还需要在动物实验中进一步证实。

综上所述，本研究结果发现人SCLC细胞株SBC3表达M3R，M3R的拮抗剂4-DAMP能抑制细胞增殖、粘附并促进细胞凋亡。我们之前的研究发现，SCLC患者癌组织较正常肺组织高表达M3R，尤其是吸烟的SCLC患者；虽然M3R和SCLC临床分期无明显相关，但M3R高表达患者的生存期明显缩短^[18]^。SCLC通常发生在年龄较大而且长期吸烟的患者身上，患者肺功能欠佳，而且很早就发生全身转移，M3R拮抗剂具有扩张支气管平滑肌、减少粘液分泌、抗气道炎症的作用，若同时能够抑制肿瘤的生长和转移，将会为SCLC尤其是同时合并慢性阻塞性肺病的患者的治疗带来新的出路。
